# Profile of children admitted with seizures in a tertiary care hospital of Western Nepal

**DOI:** 10.1186/1471-2431-13-43

**Published:** 2013-03-27

**Authors:** Sudhir Adhikari, Brijesh Sathian, Deepak Prasad Koirala, Kalipatnam Seshagiri Rao

**Affiliations:** 1 Department of Pediatrics, Manipal College of Medical Sciences, Pokhara, Nepal; 2 Department of Community Medicine, Manipal College of Medical Sciences, Pokhara, Nepal

**Keywords:** Seizures, Neurocysticercosis, Nepal

## Abstract

**Background:**

Seizure is one of the common causes of childhood hospitalization with significant mortality and morbidity. There is limited data regarding acute seizures episodes form the developing countries. Current study aims to find the common etiology of seizure and classify seizure types in various age groups presenting to tertiary center in Western Nepal.

**Methods:**

This was a hospital based retrospective study carried out in the data retrieved from the records maintained in the Department of Pediatrics, Manipal Teaching Hospital, Pokhara from 1^st^ July 2007 to 31^st^ July 2011.Variables collected were demographics, clinical presentations, laboratory tests, brain imaging studies, electroencephalography, diagnosis and hospital course.

**Results:**

A total of 551 patients were admitted for seizures with 338 (61.3%) males and 213 (38.7%) females. Among these patients, 295 (53.5%) presented with fever and 317 (57.5%) of children were less than 5 years of age. Generalized tonic-clonic seizures were the most common seizure type (69.9%). Seizure disorder (33.4%), febrile seizures (30.7%), CNS infections and neurocysticercosis were common etiologies. Abnormal brain images were noted in 111 (45.9%) of 242 patients and most common abnormality was neurocysticercosis 66 (59.5%).

**Conclusion:**

CNS infections and febrile convulsions were common causes of seizures in febrile children. Neuroimaging should be advised in all afebrile children for the diagnosis of neurocysticercosis. Children diagnosed as seizure disorder require long term follow up studies including neurophysiologic studies.

## Background

Seizures are the most common pediatric neurological disorder. Four to ten percent of children suffer at least one episode of seizure in the first 16 years of life. The incidence is highest in children less than 3 years of age, with a decreasing frequency in older children [1]. Seizures account for about 1% of all emergency department visits, and about 2% of visits of children's hospital emergency department visits [2]. The incidence of epilepsy (recurrent unprovoked seizures) in children and adolescents seems relatively consistent across all populations studied, ranging from 50 to 100/l00, 000 person-years [3]. In most of the studies, febrile seizures were reported to be the most common type seen in the pediatric population and account for the majority of seizures seen in children younger than 5 years of age [2-4].

Central nervous system (CNS) infections are the main cause of seizures and acquired epilepsy in the developing world [4,5]. Geographical variations determine the common causes in a particular region. Acute seizures are common in meningitis, viral encephalitis and neurocysticercosis and in most cases are associated with increased mortality and morbidity, including subsequent epilepsy [6-9]. The standardized mortality rate (SMR) in patients with a newly diagnosed unprovoked seizure ranges from 2.5 to 4.1 according to the study population and design. The SMR is highest in the youngest patients and in those with symptomatic seizure [10]. In most children with newly diagnosed epilepsy, the long-term prognosis of epilepsy is favorable, and in particular, patients with idiopathic etiology will eventually reach remission [11].

It is not always immediately clear which laboratory and imaging examinations should be performed when children are admitted with seizures. Children admitted in emergency department with new onset of non-febrile seizure are often evaluated using cranial computed tomography (CT) [12,13]. However, some investigations indicate brain CT scans should not be routinely arranged for these patients [14]. Treating physician have to decide for further investigations including septic screen, metabolic studies, lumbar puncture and electroencephalogram (EEG) for patients who present with a first attack of seizure. There is concern for cost of these investigations in resource poor developing countries. Misdiagnosis carries the potential risk of legal problems, can cause family anxiety, lead to excessive hospital stay, and possibly result in life-threatening events.

There are limited studies on causes and outcome acute episode of seizure in developing countries like Nepal. Most studies had done so far have focused on epilepsy and clinical seizure types [15,16]. In this retrospective study, we therefore analyzed the prevalence of various etiologies, the clinical spectrum of seizure disorders and primary outcome of children admitted with a first attack of acute seizure disorder.

## Methods

### Patient population

This was a retrospective hospital-based study conducted in the Department of Pediatrics, Manipal Teaching Hospital, Pokhara. During period of July 2007 to July 2011 a total of 6975 children in the age group 6 months to 15 years were admitted in the Pediatric Department. Among these, 551 children (12.7%) were admitted with presenting complain of seizure and included in the study. Children with seizures onset after hospitalization were excluded.

### Methods

The following information was obtained from the medical records of each patient: age (range from 6 months to 15 years), sex, type of seizure, associated symptoms (fever, cough, rhinorrhea, vomiting, diarrhea and headache), family history of seizure or epilepsy, developmental history, laboratory test results (white blood count, C-reactive protein, serum electrolytes, blood sugar and cerebrospinal fluid (CSF) analysis, neuroimaging; CT scan head or cranial magnetic resonance imaging (MRI),electroencephalography (EEG) findings, duration of hospital stay, final diagnosis. Final outcome was recorded in four categories; discharged after recovery, left against medical advice (LAMA), mortality and referral to other institutions were also recorded.

Patients were divided into two groups based on whether the seizure was with or without fever: Group 1 comprised patients with temperature recorded greater than or equal to 38°C, and Group 2 comprised patients with temperature less than 38°C. Seizure type classification, including generalized tonic-clonic (GTC), absence, myoclonic, partial and other seizures types was based on the Commission on Epidemiology and Prognosis, 1993 International League Against Epilepsy [17]. Status epileptics was defined as, “a single epileptic seizure of more than 30 minutes or a series of epileptic seizures during which function is not regained between ictal events in a period more than 30 minutes long”. Febrile seizure was defined by the 1993 International League Against Epilepsy as, “an epileptic seizure occurring in childhood after 1 month of age, associated with febrile illness not caused by an infection of the central nervous system (CNS), without previous neonatal seizure or previous unprovoked seizure, and not meeting criteria for other acute symptomatic seizure”. In addition, febrile seizures were classified as simple febrile seizures or complex febrile seizures. A simple febrile seizure lasts less than 15 minutes, is initially generalized in nature, and occurs once during a 24-hour period. In contrast, a complex febrile seizure lasts more than 15 minutes, has focal features at any time, or recurs within a 24-hour period [17].

Other etiologies including meningitis and encephalitis were diagnosed on the basis of recorded clinical and laboratory investigation and verified with standard reference [18]. Furthermore, patients were divided into three age groups: age group (6 months − 5 years), age group (6–10 years) and age group (11–15 years).Variables including age, sex, type of seizure, associated symptoms, family history of seizure or epilepsy, developmental history, laboratory test results, neuroimaging examinations, EEG findings, duration of hospital stay, diagnosis and final outcome were compared between febrile and afebrile group. These variables were also compared among children of different age groups.

### Ethical committee approval

Preceding the study, approval for the study was obtained from the institutional research ethical committee of Manipal College of Medical Sciences.

### Sample size calculation

By conducting a pilot study of 100 children with seizures it is estimated that for 95% confidence interval and, significance level α = 5%, P = 54%, Q = 46%, allowable error = 10%, required sample size was 327. P = percentage of children admitted with seizure and fever [19].

### Data analysis

Descriptive statistics and testing of hypothesis were used for the analysis. The data was analyzed using Statistical Package for the Social Sciences (SPSS) for Windows Version 16.0 (SPSS Inc; Chicago, IL, USA). The Chi-square test was used to examine the association between different variables and strength of the relationship with logistic regression. Odds ratios (OR) and their 95% confidence intervals (95% CI) were calculated. P < 0.05 was considered as statistically significant [20,21].

## Results

### Demographics, clinical seizure types in children with seizure

Table 1: There were a total of 6975 patients admitted to the ward beyond 6 month of age during the study period. Out of these patients 551(12.7%) children had seizures as a presenting complains. Among 551 children 317(57.5%) were in the age group 6 months to 5 years and was associated with fever in 232 (73.1%) of cases (p < 0.001). Fever was present on admission in 295(53.5%) of children. Afebrile seizure was common 94(80.3%) in age group 11 to 15 years. There were 338(61.3%) males and 213 (38.7%) females with male to female ratio of 1.58:1(p = 0.003). Generalized tonic clonic seizures were the commonest seizure type in this study 385 (69.9%) and 243(63.1%) of them were febrile (p < 0.001). These were followed by partial seizure 109 (19.8%), absence 13(2.7%), myoclonic 7(1.3%). Other seizures types including tonic, atonic comprised remaining 35(6.4%) of cases. Status epileptics was present in 40(7.3%) of children.

**Table 1:** Demographic data of patients presenting with seizure

	**No fever**	**Fever**	**Total**	**Odds ratio (95%-CI)**	**p**
	**n = 256(%)**	**n = 295(%)**	**n = 551(%)**		
Sex					
Male	140(54.7)	198(67.1)	338(61.3)	1.691(1.119,2.390)	0.003†
Female	116(45.3)	97(32.9)	213(38.7)	1	
Age					
6mo-5 yr	85(33.2)	232(78.6)	317(57.5)	11.15(6.637,18.749)	0.001†
6-10 yr	77(30.1)	40(13.6)	117(21.2)	2.123(1.171,3.849)	0.01†
11-15 yr	94(36.7)	23(7.8)	117(21.2)	1	
Type of seizure					
GTC	142(55.5)	243(82.4)	385(69.9)	2.896(1.415,5.927)	0.004†
Partial	72(28.1)	37(12.5)	109(19.8)	0.870(0.394,1.920)	0.730*
Absence	13(5.1)	2(0.7)	15(2.7)	0.260(0.051,1.341)	0.108*
Myoclonic	7(2.7)	0	7(1.3)	-	-
Others	22(8.6)	13(4.4)	35(6.4)	1	-
Status	21(52.5)	19(47.5)	40(7.3)	1.298(0.681,2.473)	0.428*

†Statistically significant * Not significant GTC-Generalized tonic- clonic seizure.

### Analysis of patients based on age groups

**Figure 1:**
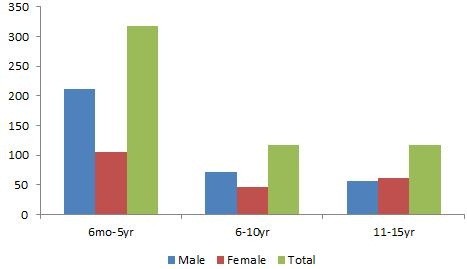
Age and sex distribution of children with seizures.

Figure 1; Seizures were more common in males in age group 6 months to 5 years 211(66.4%) and 6 to 10 years 71(60.7%). However, in the age group 11 to 15 years it was found slightly more common in females 61(52.1%).

Table 2: Lumbar puncture was performed in 317(57.5%) children with abnormal reports in 82(25.9%). CSF was reported abnormal in 42 (19.4%) of children in the age group 6 months to 5 years. Neuroimaging was done in 242 (43.9%) children admitted with seizure and included 41(56.2%) children in age group11 to 15 years. Neuroimaging had revealed abnormalities in 111(45.9%) and most common finding was neurocysticercosis in 66 children. Electroencephalogram (EEG) was done in 354(64.2%) children and had abnormal reports in 194 (54.8%) of cases. EEG abnormality was more compared to younger age in age group11 to 15 years 65(77%).

**Table 2:** Analysis of patients with seizure based on age groups

	**6 mo-5 yr**	**Age groups 6–10 yr**	**11-15 yr**	**Total**	**P value**
	**n(%)**	**n(%)**	**n(%)**	**n(%)**	
Sex					
Male	211(66.4)	71(60.7)	56(47.9)	338(61.3)	0.002†
Female	106(33.4)	46(39.3)	61(52.1)	213(38.7)	
CSF analysis					
Normal	175(80.6)	35(58.3)	25(62.5)	235(74.1)	0.001†
Abnormal	42(19.4)	25(41.7)	15(37.5)	82(25.9)	
Brain image					
Normal	65(63.7)	34(50.7)	32(43.8)	131(54.1)	0.001†
Abnormal	37(36.3)	33(49.3)	41(56.2)	111(45.9)	
Electroencephalography (EEG)					
Normal	82(53.2)	36(38.7)	32(33)	160(45.2)	0.001†
Abnormal	72(46.8)	57(61.3)	65(77)	194(54.8)	
Diagnosis					
Febrile Seizure	168(53)	-	-	168(30.5)	0.001†
Seizure disorder	69(21.8)	56(47.9)	60(51.3)	185(33.6)	
Neurocysticercosis	11(3.5)	23(19.7)	32(27.4)	66(12)	
Meningitis	19(6)	11(9.4)	6(5.1)	36(6.5)	
Encephalitis	21(6.6)	11(9.4)	5(4.3)	37(6.7)	
Cerebral Palsy	5(1.6)	3(2.6)	2(1.7)	10(1.8)	
Tubercular meningitis	3(1)	3(2.6)	2(1.7)	8(1.5)	
Hypertensive encephalopathy	1(0.2)	5(4.3)	2(1.7)	8(1.5)	
Others	20(6.3)	5(4.3)	8(6.8)	33(6)	

†Statistically significant.

**Figure 2:**
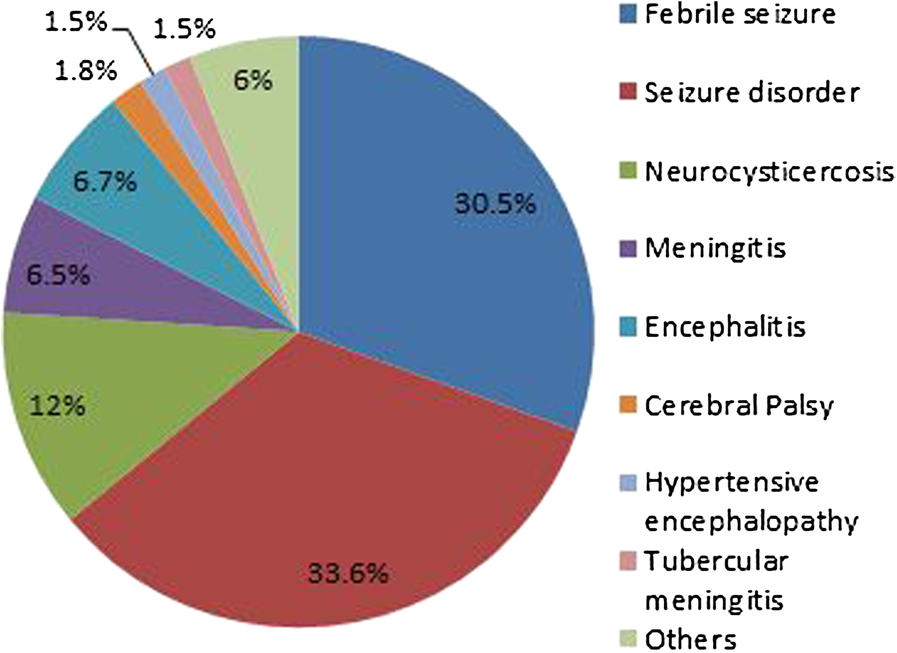
Etiological diagnosis of children with seizures.

Figure 2: Childhood seizure disorder was commonest diagnosis 185(33.6%) followed by febrile seizures 168 (30.5%), neurocysticercosis 66 (12%), meningitis 36 (6.5%) and encephalitis 37 (6.7%).

Other diagnosis made were cerebral palsy 10 (1.8%), tubercular meningitis 8 (1.5%), hypertensive encephalopathy 8 (1.5%).Miscellaneous etiologies including electrolyte imbalance (hypoglycemia, hypocalcaemia), hydrocephalus, neurocutaneous syndrome, intracranial hemorrhage, brain abscess, congenital malformations of central nervous system, hepatic and enteric encephalopathy accounted for remaining 33 (6%) of cases.

### Outcome variables in different age group and relation to fever, gender, status and diagnosis

Table 3: Final outcome was noted as discharge, death during hospital stay, left against medical advice and those referred to other specialty center for further management. Twenty four (4.4%) of children died in hospital, 22 (4%) had left against medical advice, 10 (1.8%) cases were referred and remaining were discharged after successful treatment. There was an insignificant difference in outcome between male and female, those with or without fever. Among 40 children with status epileptics only 24 (60%) were discharged from the hospital (p < 0.001). Most children with diagnosis of neurocysticercosis (98.5%) and febrile seizure (97.6%) were discharged after recovery. Children diagnosed as encephalitis and tubercular meningitis had high mortality rate only 56.8% and 75% were discharged respectively.

**Table 3:** Outcome in relation to gender, fever, status and diagnosis

	**Discharged**	**LAMA**	**Died**	**Referred**	**P value**
	**n(%)**	**n(%)**	**n(%)**	**n(%)**	
Sex					
Male	308(91)	8(2.5)	17(5)	5(1.5)	NS*
Female	187(87.8)	14(6.6)	7(3.3)	5(2.3)	
Fever					
Present	263(89.2)	9(3.1)	17(5.8)	6(2)	NS*
Absent	232(90.6)	13(5.1)	7(2.7)	4(1.6)	
Status					
Present	24(60)	5(12.5)	9(37)	2(5)	0.0001†
Diagnosis					
Febrile seizure	164(97.6)	3(1.8)	1(0.6)	-	0.0001†
Seizure disorder	169(91.4)	8(4.3)	6(3.3)	2(1.0)	
Meningitis	31(86.1)	3(8.3)	2(5.6)	-	
Encephalitis	21(56.8)	4(16.8)	9(24.3)	3(8.1)	
Neurocysticercosis	65(98.5)	1(1.5)	-	-	
Tubercular meningitis	6(75)	1(12.5)	1(12.5)	-	
Cerebral palsy	8(80)	1(10)	-	1(10)	
Hypertensive encephalopathy	8(100)	-	-	-	
Others	23(69.7)	1(3.0)	5(15.2)	4(12.1)	
Total					
551(100%)	495(89.8)	22(4.0)	24(4.4)	10(1.8)	

†Statistically significant * Not significant.

## Discussion

This was a hospital based retrospective study of children admitted with acute episode of seizure in a tertiary care center in the western region of Nepal from July 2007 to July 2011. It aimed in studying demographics, clinical seizure types, etiologies and outcome during the hospital stay of those children. Neonates and infants under 6 months of age were excluded from the study because frequently they have conditions like septicemia, hypoxic-ischemic encephalopathy, metabolic disorders which comprise one spectrum of diseases [22].

### Demographics and clinical seizure types

Most studies show high incidence of seizures in younger children with a decreasing frequency in older age group and more common in males [2,5]. Most children with seizures in our retrospective study were younger than 5 years of age. Males had higher prevalence compared to female in age group less than 10 years. Strikingly higher prevalence in female was noted in age group more than 10 years. Seizures coexisted with fever in 53.5% of cases. Most studies show generalized seizures are much more common compared to partial seizure [4,5,7]. In the current study generalized tonic-clonic was commonest seizure type and found to have higher incidence among febrile children. Partial seizure was common among children of developing countries with the setting of high incidence of neurocysticercosis [8]. Partial seizures represented only 109(19.8%) of children in the current study.

### Etiological profile

Whether routine neuroimaging should be done in all children admitted with acute episode of seizure is debated [5,12]. In this study abnormal neuroimaging was present in 111 (45.9%) and showed abnormal CT was found more in older afebrile children. There seems role of routine neuroimaging in afebrile children with seizures in age group more than 5 years in developing countries with high prevalence of neurocysticercosis. AAP recommends lumbar puncture for febrile seizure children aged less than 12 months [22]. CSF abnormality was more in children of age groups more than 5 years compared to younger age group. Lumbar puncture may be done in selected children guided by physical finding to rule out CNS infections older children. There are many possible etiologies of a first seizure attack in children, including infection, neurologic/developmental causes, traumatic head injury, toxins, and metabolic disturbances [4-6]. Febrile seizures have been reported to be one of the most common causes of seizure attack in children [2-4]. We found that febrile seizures (53.0%) were the main etiology of a first attack of seizure in children less than 5 years of age. Overall, seizure disorder was commonest etiology in children aged 6 months to 15 years (33.6%) followed by febrile seizure (30.5%).

### Primary outcome of acute seizure

gMortality rate during hospital course among children admitted with acute episode of seizure was similar with reports from other developing countries [4]. There was no significant difference in the outcome among male and female. Fever was not independently associated with increased mortality during the acute illness. Meningitis and encephalitis cause significant childhood mortality and morbidity [4,6]. Children with diagnosis of encephalitis and those with status epileptics had poor outcome with high mortality [23]. Febrile seizure, neurocysticercosis and hypertensive encephalopathy had good outcome with majority of children discharged after recovery.

As evident from current study provoked seizures including CNS infections and neurocysticercosis account for majority of cases. Most of these might be prevented with improvement in sanitation. Since 2009 there is routine immunization for Hemophilus influnzae b and Japanese encephalitis vaccine was introduced in selected districts of Nepal [24]. Attempt should be made to know the burden of other causative organisms for CNS infections and preventive measures should be undertaken. Health care facilities should be prepared for emergency management of seizures to decrease mortality and morbidity.

### Limitations of the study

Outcome was defined as mortality during hospital stay and we were unable to study morbidities like neurological dysfunction and impact on scholastic performance. The details of other causes contributing for seizures like inborn error of metabolism could not be specified due lack of investigations. Multi centric prospective study is needed to find out details regarding these problems.

## Conclusion

Acute episode of seizures are one of the commonest cause of hospitalization with high mortality. It can be made from our study that most of acute symptomatic seizures are caused by febrile seizures, CNS infections like meningitis and encephalitis, neurocysticercosis which can be prevented with improvement in health care facilities. Group of children presenting with unprovoked seizure require long term follow up studies including neurophysiologic studies and neuroimaging (CT or MRI) for better understanding of childhood seizure disorder in developing countries contest.

## Competing interests

The authors do not have any conflict of interest arising from the study.

## Authors’ contributions

SA and KSR designed the study, deduced the data, drafted the manuscript, and revised it. BS planned the study with SA, conducted the data analysis, interpreted the data, and revised the manuscript. KSR and DPK critically revised the manuscript. All the authors approved the final document.

## Pre-publication history

The pre-publication history for this paper can be accessed here:

http://www.biomedcentral.com/1471-2431/13/43/prepub

## What this study adds

This study provides information about causes, clinical seizures types and outcome of children admitted with seizures in Western region of Nepal.
